# Klipple-Trenaunay Syndrome: A Rare Disorder With Multisystemic Clinical Attributes

**DOI:** 10.7759/cureus.19776

**Published:** 2021-11-20

**Authors:** Uma Gupta, Prasenjit Sarker, Tutul Chowdhury

**Affiliations:** 1 Internal Medicine, Chittagong Medical College, Chittagong, BGD; 2 Internal Medicine, One Brooklyn Health System, Brooklyn, USA

**Keywords:** soft tissue malformation, skin discoloration, klipple-trenaunay syndrome, port-wine stain, recurrent gi bleeding, pik3ca gene, bone or soft-tissue growth, capillary venous malformation

## Abstract

Klippel-Trenaunay syndrome (KTS) is a rare disorder characterized by abnormal development of soft tissues, lymphatic system, and blood vessels. Major features include tissue and bone overgrowth, vein malformation, and port-wine stains with or without lymphatic abnormalities. It is crucial to review this rare syndrome to avoid any diagnostic delay. In addition, it is also vital to follow disease courses with symptomatic treatment for rare complex diseases, which would help clinicians understand and implement a better treatment plan in the future. We present the case of a 19-year-old male eventually diagnosed with KTS who initially presented with swelling of his feet and skin erosion with bloody discharge. Associated findings were bluish skin discoloration, nodularity, and bleeding per rectum, leading to anemia and subsequent heart failure. Colonoscopy/sigmoidoscopy showed vascular malformation and an active bleeding site. Our patient manifested most of the clinical attributes of KTS, with an interesting clinical course of arteriovenous, soft tissue, capillary, lymphatic, and vascular malformations. However, in our case, the patient is receiving only symptomatic treatment (blood transfusion) without any limb amputation or reconstruction surgery, leading to no further deterioration of the quality of life.

## Introduction

In 1900, two French physicians, Dr. Klipple and Dr. Trenaunay, described a rare congenital condition of capillary-venous-lymphatic-soft tissue malformation and named it Klippel-Trenaunay syndrome (also known as KTS). KTS is a complex genetic disorder of random mosaic mutation during the in-utero cell division process that activates the *PIK3CA* gene (phosphatidylinositol-4,5-bisphosphate 3-kinase catalytic subunit alpha gene) [1]. In addition, the gain of function mutation in *PIK3CA* causes activation in mammalian targets of rapamycin (mTOR) and protein kinase B, which is responsible for cell proliferation and angiogenesis [2,3]. The International Society of vascular anomalies further outlines the definition of KTS in 2018, where a triad of capillary malformation, venous malformation, and limb overgrowth with or without lymphatic involvement coexists [4].

There is no available information regarding the incidence and prevalence of this condition [5]. Therefore, the treatment and management varied significantly from case to case at providers' discretion [5]. In the last 39 years, there have been 239 cases of KTS in Mayo Clinic, yet there is no available curative treatment for it. Recurrence following surgical management is common, and limited non-surgical treatment options are available. In our case, the patient had developed arteriovenous malformation later in the course of the disease. However, our patient was diagnosed with KTS 18 years ago when he presented with a triad of capillary malformation, venous malformation, bone or soft tissue growth, and relevant complications.

## Case presentation

A 19-year-old male has presented to a tertiary hospital with per rectal bleeding for the last five years. He complained of frequent bright red per rectal bleeding after defecation. The patient has been given a history of shortness of breath, fatigue, and occasional chest pain for the last two months. In addition, there is a recent history of multiple hospitalizations for anemia associated with per rectal bleeding with subsequent heart failure. In the previous six months, he received 12 units of blood transfusion. He did not have any fever, diarrhea, nausea or vomiting, weight loss, abdominal pain, anal or rectal pain, any previous history of abdominal surgery, a blood disorder, or radiation therapy.

**Figure 1:**
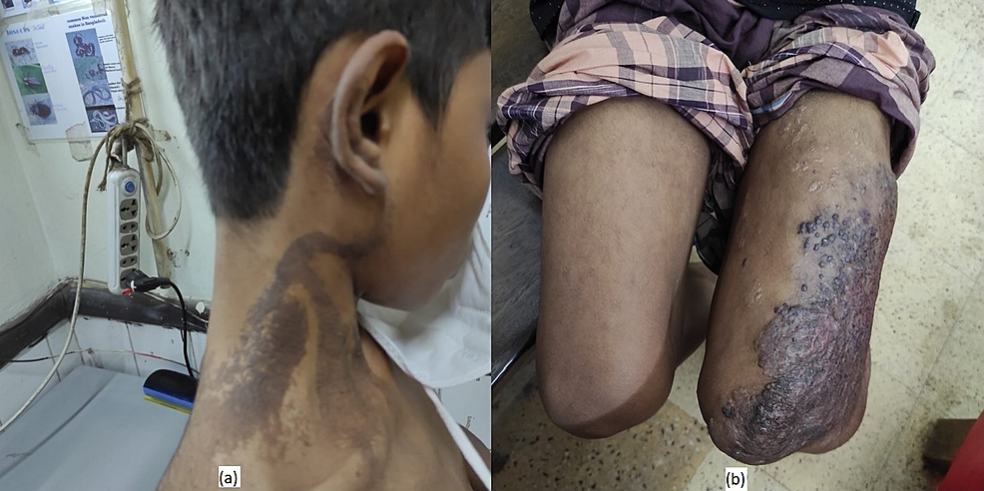
(a) Port-wine stain over the nape of the neck. (b) Overlying skin change of vascular malformation.

He was born with normal vaginal delivery after uncomplicated labor, and his mother also had an uneventful pregnancy with a periodic antenatal check-up. Post-birth follow-up shows a fluid-filled sac over the right occipital and right lower rib cage, which was later treated with fluid aspiration and sclerotherapy at three months. In addition, there was left leg swelling since birth with a port-wine stain over the nape of his neck (Figure 1a), upper arm, and upper back. He had a history of easy bleeding from his lower limb swelling at his toddler age, which needed emergency service assistance several times. However, he did not mention any difficulties in walking or cognitive function during his childhood.

**Figure 2:**
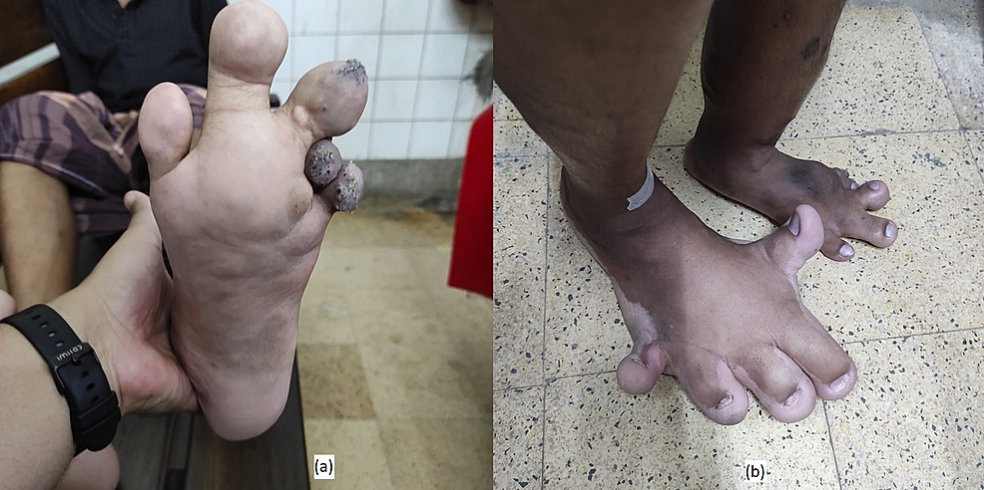
(a) Deformities of toes with skin erosions. (b) Lymphatic, soft tissue swelling, and vascular malformation of the lower limb.

At the age of six years, he developed fatigue, and there was foul-smelling bloody discharge from his skin eruption over his left thigh and left side of the body. When he was nine years old, his skin lesions were progressively worsening, initially at his neck and then mid-thigh with irregular bluish skin discoloration and a papular lesion (Figure 1b). He had no pruritus but reported occasional bloody discharge with minor trauma. Later at the age of 12 years, he noticed significant gradual soft tissue swelling in his left foot (Figure 2a) and toes with overlying skin erosions, easy bleeding, and ulceration (Figure 2b). Eventually, at the age of 15 years, he developed per rectal bleeding, which was mild at the beginning and then gradually worsened in the last two years.

The chronological order of the patient’s disease is given in Table 1.

**Table 1:** Chronological order of disease description.

Age	Events
At birth	Fluid-filled sac over the right occipital and right lower rib cage; port-wine stain over the nape of his neck, upper arm, and upper back; left leg soft tissue swelling
1-3 years	Skin erosion with bloody discharge from his lower limb swelling
3-6 years	Foul-smelling bloody discharge from his skin eruption over his left thigh and left side of the body
6-9 years	Irregular bluish skin discoloration and a papular lesion with gradual deterioration
9-12 years	Soft tissue swelling in his left foot and toes with overlying skin erosions; easy bleeding and ulceration of the skin erosion
12-15 years	Gradual worsening of limb swelling; delayed puberty; Per rectal bleeding
15-19 years	Per rectal bleeding; severe Anemia; heart failure

We learned from collateral history that he was bullied for his appearance and deformities in his school, was a non-smoker and non-alcoholic, and has no relevant family history.

**Figure 3:**
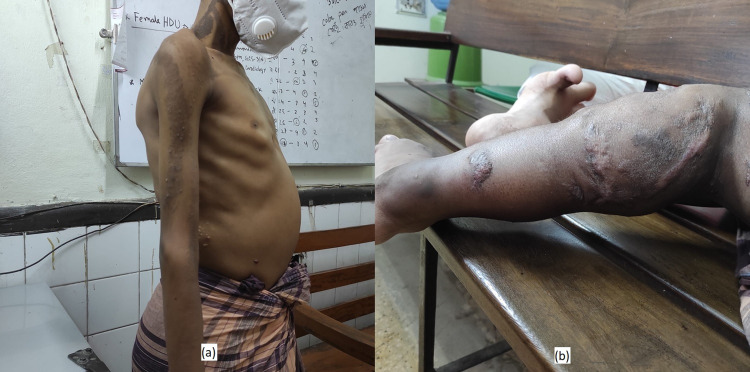
(a) Malnourished built with a papular skin lesion and brownish skin discoloration. (b) Scarring and irregular skin lesion due to underlying lymphatic and vascular malformation.

The patient was found malnourished (Figure 3a), pale with shortness of breath, and sleeping in an upright position on his bed. He had brownish discoloration of skin at the nape of his neck (approximately 10 x 12 cm) with a widespread papular skin lesion over his back, neck, and upper limb (Figure 3a). In addition, there is approximately 12 x 14 cm bluish discoloration with nodular deformities of the skin over the left calf (Figure 3b).

The patient was atraumatic and normocephalic without any lymphadenopathy. Vitals were stable with regular S1 and S2, S3 was appreciated, and bilateral basal crepitations were present on lung auscultation. All peripheral pulses were palpable. His abdominal examination findings revealed a distended abdomen with no tenderness, no mass, or organomegaly. On evaluation, his secondary sexual characteristics were not well developed. After further examination, we found that his genitalia, axillary, and pubic hair have been arrested in Tanner stage 2 for the last five years. The patient looked like a 10-year-old boy with no voice changes and sexual development. We also found swelling and firm mass in his scrotum and genitalia.

**Figure 4:**
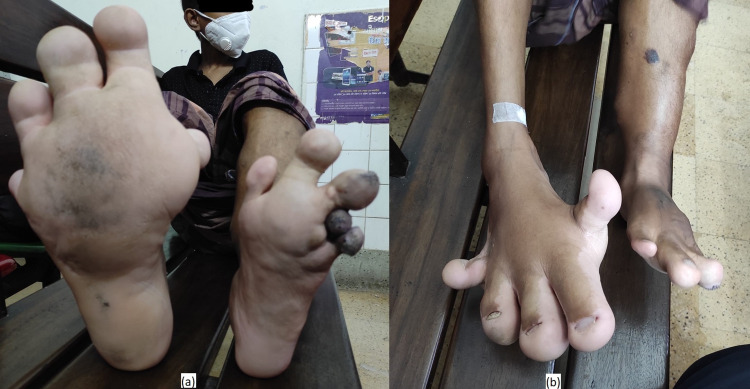
(a) Deformities of the toes. (b) Structural abnormalities and feet deformities.

Left toes were swollen due to soft tissue swelling (Figures 4a, 4b). However, his gait was normal. Muscle power was 5/5, muscle tone was normal, there was no sensory loss, and focal neurological deficits were not present. Reflexes of all four limbs were normal.

He is taking no other medication except as-needed pain medication and multivitamins. He is not allergic to any medicines, food, or environmental triggers.

On his recent laboratory examination, his hemoglobin was 3.7 g/L, which has improved to 7.8 g/L after four units of blood transfusion. Peripheral blood film has confirmed microcytic hypochromic anemia, most likely due to iron deficiency anemia (ferritin: 9.56 ng/mL; serum iron: 105 ug/dL; total iron-binding capacity: 370 ug/dL). Other lab tests such as activated partial thromboplastin time (aPTT), prothrombin time (PT), fibrinogen, and fibrin degradation products were normal, with no intravascular coagulopathy features. Chest X-ray and EKG were within normal limits, with 55% ejection fraction on a recent echo. On the other hand, his exercise tolerance test was limited for shortness of breath and fatigue. In addition, his autoimmune panel (anti-cyclic citrullinated peptide [anti-CCP] and anti-double stranded DNA [anti-dsDNA]), liver function test, and kidney function test were all within the average level. A recent colonoscopy confirmed arteriovenous malformation over the left colon. In addition, a color Doppler study of the lower limb revealed venous anomalies.

**Figure 5:**
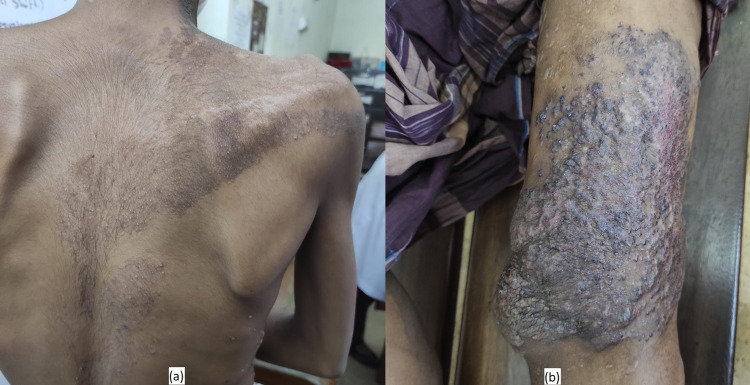
(a) Brownish skin discoloration of the back. (b) Localized bluish discoloration of the skin of the lower limb.

Our patient developed shortness of breath on physical exertion, limiting his mobility and transfer from bed to chair. In addition, there was port-wine skin discoloration of his back (Figure 5a) and localized bluish skin color changes with nodularity (Figure 5b). Unfortunately, his parents were paying his medical expenses, and resources were limited. The next plan of further management is bowel reconstruction to treat his per rectal bleeding by repairing arteriovenous malformation of the left colon. He had undergone extensive sclerotherapy and imaging treatment without any genetic analysis, with no apparent improvement. Unfortunately, there is no recent MRI or CT scan of the abdomen at this point.

## Discussion

KTS without lymphatic involvement is diagnosed as diffuse capillary malformation with overgrowth (DCMO), which presents with more proportionate overgrowth and diffuse or reticulate stains [5]. The mosaic mutation can occur at any stage of in utero developmental process, and the severity of KTS depends on the timing of this mutation during this developmental stage. If this mutation occurs early in the developmental stage, it will cause severe disease expression after birth. At birth, patients usually present with a vascular stain (port-wine stain), which is geographic or blotchy/segmental, dark-red, purple, or pink-red [6]. The skin discoloration is also proportionate to disease severity. Venous varicosities in the anterolateral thigh and leg are usually present at birth or in infancy due to the abnormal embryonic avalvular venous structure [7]. In childhood, the lymphatic malformation presents with chronic leakage of lymph or blood or secondary infection with pseudoverrucous hyperplasia and lymphedema, which leads to enlargement of the affected limb. The disfigurement of the genitalia can happen due to deep lymphatic malformation in the pelvis and retroperitoneal region [8]. The reasons for limb overgrowth could be multifactorial, such as soft tissue and bone overgrowth with or without vascular and lymphatic malformation [8]. There is a cohort study of 48 people in which 23 (48%) patients presented with superficial venous thrombosis, 16% (8 among 48 people) developed deep vein thrombosis and pulmonary embolism, and 2% people developed pulmonary embolism [9].

Laboratory abnormalities usually present with low platelet, low fibrinogen, and elevated D-dimers; however, in our case, levels are all within normal limits except mild thrombocytopenia. Bleeding from vascular anomalies and ectasia is common, especially gastrointestinal (GI) bleeding (with the most common site being rectum) due to intra-pelvic venous anomalies and varicosities [10]. Other complications are limb length discrepancy, lipodermatosclerosis, stasis dermatitis, and recurrent bouts of cellulitis. Pain is another debilitating and multifactorial symptom due to venous insufficiency, intra-articular involvement (such as hemarthrosis), and neuropathic pain [11]. Imaging modalities such as ultrasonography can diagnose venous anomalies. MRI can be used for diagnostic accuracy, which is also used to diagnose lymphatic abnormalities and bone and soft tissue growth. Somatic mutation of involved tissue can be diagnosed by PCR (polymerase chain reaction). However, a definite diagnosis needs single-molecule molecular inversion probes [12]. Contrast venography is a helpful tool before and after endovascular and intralesional treatment of venous malformation [13]. Other available treatment options are compression therapy, laser therapy, physical therapy, sclerotherapy, and surgery. It was evident from recent research that Sirolimus (mTOR inhibitors) and Alpelisib (*PIK3CA* inhibitors) could be helpful to a limited extent [14]. Low-dose aspirin can help in a patient with venous malformation to decrease the chance of thrombosis. However, no definite treatment is yet established.

## Conclusions

KTS is a rare and debilitating disease. Our patient developed port-wine stain, soft tissue swelling, and localized vascular malformation from birth. He also had a history of bloody discharge from skin lesions and rectal bleeding, which led to anemia and heart failure. Although the disease course is progressive, in his case, regular blood transfusion for GI bleeding is a symptomatic treatment option for several years. This is a unique case to review as the patient did not undergo any reconstructive surgery yet; however, he is maintaining his health condition with periodic blood transfusions.
